# Coordinated reset stimulation in a large-scale model of the STN-GPe circuit

**DOI:** 10.3389/fncom.2014.00154

**Published:** 2014-11-27

**Authors:** Martin Ebert, Christian Hauptmann, Peter A. Tass

**Affiliations:** ^1^Institute of Neuroscience and Medicine - Neuromodulation, Juelich Research Center GmbHJuelich, Germany; ^2^Department of Physics, Institute of Nuclear Physics, University of CologneCologne, Germany; ^3^Department of Neurosurgery, Stanford UniversityStanford, CA, USA; ^4^Department of Neuromodulation, University of CologneCologne, Germany

**Keywords:** deep brain stimulation, high-performance computing, coordinated reset stimulation, electrode design

## Abstract

Synchronization of populations of neurons is a hallmark of several brain diseases. Coordinated reset (CR) stimulation is a model-based stimulation technique which specifically counteracts abnormal synchrony by desynchronization. Electrical CR stimulation, e.g., for the treatment of Parkinson's disease (PD), is administered via depth electrodes. In order to get a deeper understanding of this technique, we extended the top-down approach of previous studies and constructed a large-scale computational model of the respective brain areas. Furthermore, we took into account the spatial anatomical properties of the simulated brain structures and incorporated a detailed numerical representation of 2 · 10^4^ simulated neurons. We simulated the subthalamic nucleus (STN) and the globus pallidus externus (GPe). Connections within the STN were governed by spike-timing dependent plasticity (STDP). In this way, we modeled the physiological and pathological activity of the considered brain structures. In particular, we investigated how plasticity could be exploited and how the model could be shifted from strongly synchronized (pathological) activity to strongly desynchronized (healthy) activity of the neuronal populations via CR stimulation of the STN neurons. Furthermore, we investigated the impact of specific stimulation parameters especially the electrode position on the stimulation outcome. Our model provides a step forward toward a biophysically realistic model of the brain areas relevant to the emergence of pathological neuronal activity in PD. Furthermore, our model constitutes a test bench for the optimization of both stimulation parameters and novel electrode geometries for efficient CR stimulation.

## 1. Introduction

Several brain disorders are associated with abnormal neuronal synchronization. For example, epileptic seizures are characterized by an abnormally synchronized firing of neurons during the epileptic process (Lehnertz et al., 2009). Parkinson's disease (PD) is another pathological condition of the central nervous system, which is associated with abnormal neuronal synchronization in specific brain regions (Lenz et al., 1994; Nini et al., 1995; Plenz and Kital, 1999). The occurrence of resting tremor with frequencies between 4 and 9 Hz in PD is correlated to pathological synchronization of particular neuronal populations located in the thalamus and basal ganglia (Deuschl et al., 2000; Bevan et al., 2002; Mallet et al., 2008; Smirnov et al., 2008; Tass et al., 2010), which actually seem to drive the tremor (Smirnov et al., 2008; Tass et al., 2010). In contrast, in the healthy state these populations fire in an uncorrelated manner (Nini et al., 1995; Brown et al., 2001; Fahn, 2008).

An established therapy for patients with medically refractory PD is electrical deep brain stimulation (DBS) (Benabid et al., 1991; Rodriguez-Oroz et al., 2005; Deuschl et al., 2006). Today the field of application of DBS is expanding. For instance, DBS has recently been evaluated in clinical trials in patients suffering from treatment-resistant depression, and first-in-man studies have been conducted for the treatment of Tourette's syndrome (Anderson et al., 2012; Cannon et al., 2012). Despite positive clinical results, the understanding of the mechanisms of action underlying the therapeutic effects of DBS is still a matter of debate (Grill and McIntyre, 2001; Volkmann, 2004; Anderson et al., 2012).

For DBS depth electrodes are implanted in particular target areas during high precision stereotactic surgery (Voges et al., 2002; Coffey, 2009). For the treatment of PD, the STN is the most commonly used target for DBS (Rodriguez-Oroz et al., 2005; Deuschl et al., 2006). This small lens-shaped brain structure is part of the basal ganglia, which are important for voluntary movement control (Nini et al., 1995). The electrodes mostly used for implantation comprise four cylindrical contacts, which are made of a Pt-Ir alloy. Each contact can individually be activated for stimulation (Coffey, 2009). Extension cables connect the electrodes to the pacemaker, which is implanted below the patient's collarbone and serves as counter-pole during stimulation. The standard DBS algorithm is high-frequency (HF) permanent stimulation, where a biphasic current pulse train is continuously administered via the implanted electrode contact(s). Stimulation frequencies of 100 − 180 Hz and amplitudes varying between 0 and 10 V have clinically proven to be effective (Moro et al., 2002; Volkmann et al., 2002). The mechanisms of DBS are still matter of debate (McIntyre et al., 2004c). One hypothesis is, that HF DBS leads to a suppression of activity in the target structure and thus mimics lesioning effects in a reversible way (McIntyre et al., 2004c; Volkmann, 2004; Kühn et al., 2008; Montgomery and Gale, 2008; Pyragas et al., 2013). However, there is evidence for other non-local mechanisms (McIntyre et al., 2004c; Gradinaru et al., 2009). Rubin et al. (2012) gives a good review of the complex mechanisms of DBS based on experimental and numerical observations.

HF DBS treatment allows for a significant reduction of medication (Deuschl et al., 2006). However, there is a relevant non-responder rate, for instance, tremor suppression is non-effective in 15% of the patients, and in 73% of the patients HF DBS is non-effective with respect to axial symptoms (Limousin et al., 1999; Volkmann, 2004). In fact, not all symptoms are equally reduced (Rodriguez-Oroz et al., 2005), some patients show side effects such as worsening speech, reduced verbal fluency, and weight gain affect an important number of patients (Voges et al., 2002). Some of these side effects may result from current spread to remote structures or improper electrode placement, but the underlying reasons for the occurrence of side effects are still not sufficiently understood (Voges et al., 2002).

To overcome limitations of HF DBS, with methods from non-linear dynamics and statistical physics, stimulation techniques have been developed, which specifically counteract pathological neuronal synchronization by desynchronization (Tass, 1999). Coordinated reset (CR) stimulation is a robust desynchronization technique which can be applied in an open loop as well as closed loop mode (Tass, 2003b,c).

CR aims at a therapeutic reshaping of neuronal connectivity by harnessing synaptic plasticity (e.g., spike timing-dependent plasticity, STDP) (Gerstner et al., 1996; Markram et al., 1997). Based on Shen et al. (2003) we hypothesize that STDP also occurs within the STN population. The goal of CR stimulation is to decrease the rate of coincidences, which – due to STDP – causes a decrease of the synaptic weights and, in turn, an unlearning of pathological connectivity and synchrony (Tass and Majtanik, 2006). Put otherwise, CR DBS aims at long-lasting effects that persist after cessation of stimulation (Tass and Majtanik, 2006; Tass and Hauptmann, 2007). First experimental *in vitro* studies in rat hippocampal slice and *in vivo* experiments in Parkinsonian monkeys confirmed that CR causes a long-lasting desynchronization (Tass et al., 2009) and reduction of symptoms (Tass et al., 2012b). By the same token, long-lasting and cumulative aftereffects of CR stimulation were observed in a proof of concept study in PD patients (Adamchic et al., 2014a). Computational studies showed that CR stimulation can be delivered both invasively and non-invasively (Popovych and Tass, 2012; Tass and Popovych, 2012). Accordingly, CR has successfully been applied to the treatment of tinnitus via acoustic stimulation (Tass and Popovych, 2012; Tass et al., 2012a; Silchenko et al., 2013; Adamchic et al., 2014b).

The biophysical mechanism of action of DBS is not yet sufficiently understood (Grill and McIntyre, 2001; Volkmann, 2004). In order to investigate how electrical stimulation affects neuronal tissue, computational models of the respective systems are useful tools (Rubin and Terman, 2004; Miocinovic et al., 2006; So et al., 2012). In the present study we focus on the effects of different electrical stimulation algorithms on the collective activity of a model neuronal network. So far, the mechanisms of CR stimulation have been studied utilizing neuron models with reduced complexity and a limited number of modeled neurons (Hauptmann et al., 2005; Tass and Hauptmann, 2009; Guo and Rubin, 2011; Lysyansky et al., 2011). Therefore, in this study we consider a sufficient number of neurons required to appropriately sample the stimulated volume in space and establish a computational platform for time consuming simulations taking into account the slow STDP-mediated dynamics.

The high number and density of neurons gave us the opportunity to investigate local and propagation effects of synchronization within the network. Hence, we constructed a large-scale model of the two structures hypothesized to be responsible for the pathological activity associated with PD. Our model contains a network of in total 2 · 10^4^ neurons. In contrast to previous models, where the nodes of the simulated networks were placed on one- or two-dimensional (regular) lattices (Hauptmann et al., 2005; Guo and Rubin, 2011; Lysyansky et al., 2011), and similar to a previous study (Hauptmann and Tass, 2010) we arranged the neurons within a spatial network in order to replicate the three-dimensional structure of the simulated parts of the brain. We used a conductance based, and biophysically realistic model associated with physical dimensions for the individual neurons (Terman et al., 2002; Rubin and Terman, 2004).

With increased complexity, however, it becomes challenging to fully understand the dynamics of the system. Hence, we mainly based our model on experimentally constrained parameters and continued the top-down approach of previous studies (Hauptmann et al., 2005; Hauptmann and Tass, 2007; Maistrenko et al., 2007; Tass and Hauptmann, 2007, 2009; Hauptmann and Tass, 2009, 2010; Lysyansky et al., 2011; Popovych and Tass, 2012) by increasing the complexity of the considered model gradually. The model presented in this study can be considered as a step forward toward a biophysically realistic model of a major target area for DBS.

## 2. Methods

We used *Neural Simulation Tool (NEST) (version 2.0.0-rc4)* (Gewaltig and Diesmann, 2007) to implement and perform the simulations presented in this study. The simulation code of the model network was implemented using the *Python* interface for *NEST* (Eppler et al., 2008). *NEST* allows for an implementation of three dimensional models of neuronal networks via the topology module (Plesser and Austvoll, 2009). We performed all simulations on the high-performance computer *JUROPA* at the Research Center Jülich, Germany. This supercomputer is composed of 3288 compute nodes, while each node comprises two *Intel Xeon X55704* quad-core processors, resulting in 26304 processors in total available for computation. The supercomputer is equipped with a main memory capacity of 79 TB and provides 274.8 FLOPS performance, measured by the LINPACK-benchmark. Accordingly, each compute node has access to 24 GB of memory. This high amount of memory available per node allows for simulations with complex individual neurons and an online analysis of simulation data. The various computation nodes are linked via an *InfiniBand* quad data rate fat tree topology. The used *Message Passing Interface (MPI)* implementation was *Parastation-MPI 5.0.25*. The differential equations describing the dynamics of our model network were solved numerically utilizing an embedded Runge-Kutta-Fehlberg method of fourth order with adaptive step size control, which is implemented as part of the *GNU Scientific Library (GSL) (version 1.14)* (Fehlberg, 1970; Galassi et al., 2009).

### 2.1. Model equations

In order to model the neuronal activity of the individual STN and GPe neurons, we used the model developed by Terman and Rubin (Terman et al., 2002; Rubin and Terman, 2004), which is a single-compartment conductance based model.

(1) $$c_{\text{m}}\frac{dv}{dt}\text{ ​}=\text{ ​}-I_{\text{L}}\text{​}-I_{\text{K}}\text{​}-I_{\text{Na}}\text{​}-I_{\text{T}}\text{​}-I_{\text{Ca}}\text{​}-I_{\text{ahp}}\text{​}-I_{\text{syn}}\text{​}+I_{\text{stim}}\text{​}+I_{\text{noise}}$$

The Na^+^ and K^+^ ionic currents *I*_Na_, *I*_K_ are responsible for the spike production. Besides those spike-producing currents a low-threshold T-type Ca^2+^ current *I*_T_, a high-threshold Ca^2+^ current *I*_Ca_ along with a K^+^ current *I*_ahp_, which kicks in after hyperpolarization, enter the equation. The intrinsic neuron dynamics is additionally influenced by synaptic inputs from connected STN and GPe neurons *I*_syn_, the input from surrounding brain structures *I*_noise_, and an external stimulation current *I*_stim_. Via this stimulation current the influence of DBS on each single neuron is modeled. The various ionic currents are all expressed in pA/μm^2^ and described by several equations:

(2) $$\;I_{\text{L}}=g_{\text{L}}\left[v-v_{\text{L}}\right]$$

(3) $$\;I_{\text{K}}=g_{\text{K}}n^{4}\left[v-v_{\text{K}}\right]$$

(4) $$\;I_{\text{Na}}=g_{\text{Na}}m_{\infty }^{3}\left(v\right)h\left[v-v_{\text{Na}}\right]$$

(5) $$\;I_{\text{T}}=g_{\text{T}}a_{\infty }^{3}\left(v\right)b_{\infty }^{2}\left(r\right)\left[v-v_{\text{Ca}}\right]$$

(6) $$\;I_{\text{Ca}}=g_{\text{Ca}}s_{\infty }^{2}\left(v\right)h\left[v-v_{\text{Ca}}\right]$$

(7) $$\;I_{\text{ahp}}=g_{\text{ahp}}\left[v-v_{\text{K}}\right]\frac{\left[\text{Ca}\right]}{\left[\text{Ca}\right]+k_{1}}$$

(8) $$\frac{d\left[\text{Ca}\right]}{dt}=\Gamma \left(-I_{\text{Ca}}-I_{\text{T}}-k_{\text{Ca}}\left[\text{Ca}\right]\right)$$

**Figure 1:**
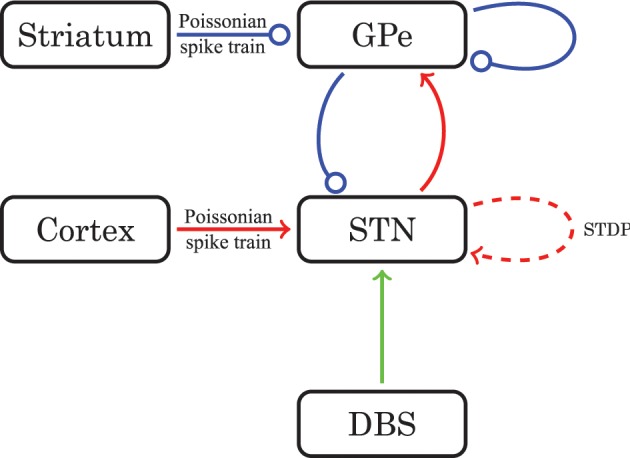
**Schematic diagram of the model network structure**. Excitatory connections are in red (with arrows) and inhibitory connections are in blue (with dots). For connections drawn as solid lines the synaptic weights are constant in time. The dashed line stands for connections governed by STDP. The green line represents optional external electrical stimulation delivered to the STN.

The equations for both STN and GPe neurons are very similar. Besides different parameter values, the only difference is the slightly simpler form of the low threshold Ca^2+^ current *I*_T_ = *g*_T_*a*^3^_∞_ (*v*) *r* [*v* − *v*_Ca_] for the GPe neurons. In our model the parameters for the reversal potentials and maximum conductances of the respective ion channels were Gaussian distributed around the mean values given in Tables 1, 2. We chose a standard deviation equal to 5% of the mean value. Thus, not all neurons were identical, which led to a more realistic scenario. A schematic diagram of our model network is illustrated in Figure 1. We focussed on a detailed description of the connections within and between the two nuclei, the STN and the GPe. Hence, our model consists of two mutually interacting sub-networks representing the STN and the GPe. The connectivity matrix of the entire network could be written as a combination of several sub-networks:

(9) $$W=\left(\begin{array}{cc} w_{\text{ss}} & w_{\text{gs}}\\ w_{\text{sg}} & w_{\text{gg}} \end{array}\right)$$

*w*_ss_, *w*_gs_, *w*_sg_, and *w*_gg_ represent the connection matrices for the STN-STN (ss), STN-GPe (sg), GPe-STN (gs), and GPe-GPe (gg) sub-networks, respectively. Since we considered the simulated neuronal network as a weighted and directed graph, the matrix was not necessarily symmetric and each entry *W*_*ij*_ corresponded to the connection strength between neuron *i* and *j*. We excluded self-connections in our model, which resulted in: *W*_*ii*_ = 0∀_*i*_.

**Table 1:** **Parameter values used for STN neurons**.

	**nS/ μm^2^**		**mV**
*g*_L_	2.25	*v*_L_	−60.0
*g*_K_	45.0	*v*_K_	−80.0
*g*_Na_	37.5	*v*_Na_	55.0
*g*_Ca_	0.5	*v*_Ca_	140.0
*g*_ahp_	9.0	*v*_ss_	0.0
*g*_T_	0.5	*v*_sg_	0.0
	**ms**		**ms**
η_*h*_	500.0	ξ_*h*_	1.0
η_*n*_	100.0	ξ_*n*_	1.0
η_*r*_	17.5	ξ_*r*_	40.0
ϑ_*h*_	0.75	ϑ_*n*_	0.75
ϑ_*r*_	0.2		
τ_ss_	1.0	τ_gs_	3.3
θ_*m*_	−30.0	*o*_*m*_	−30.0
θ_*h*_	−39.0	*o*_*h*_	−39.0
θ_*n*_	−32.0	*o*_*n*_	−32.0
θ_*r*_	−67.0	*o*_*r*_	−67.0
θ_*a*_	−63.0	*o*_*a*_	−63.0
θ_*b*_	0.4	*o*_*b*_	0.4
θ_*s*_	−39.0	*o*_*s*_	8.0
μ_*h*_	−57.0	γ_*h*_	−3.0
μ_*n*_	−80.0	γ_*n*_	−26.0
μ_*r*_	68.0	γ_*r*_	−2.2
*k*_1_	15.0	*k*_Ca_	22.5
Γ	0.0375 $\frac{\text{mol}}{\left({\text{lAs}}^{2}\right)}$	*c*_m_	1 $\frac{\text{pF}}{\mu m^{2}}$

The maximum conductances *g*_ion_ and reversal potentials *v*_ion_ were Gaussian distributed with a standard deviation of 5% around the respective mean values given in this table.

**Table 2:** **Parameter values used for GPe neurons**.

	**nS/μm^2^**		**mV**
*g*_L_	0.1	*v*_L_	−55.0
*g*_K_	30.0	*v*_K_	−80.0
*g*_Na_	120.0	*v*_Na_	55.0
*g*_Ca_	0.15	*v*_Ca_	120.0
*g*_ahp_	30.0	*v*_gg_	−80.0
*g*_T_	0.5	*v*_gs_	−100.0
	**ms**		**ms**
η_*h*_	0.27	ξ_*h*_	0.05
η_*n*_	0.27	ξ_*n*_	0.05
η_*r*_	30.0		
ϑ_*h*_	0.05	ϑ_*n*_	0.05
ϑ_*r*_	1.0		
τ_gg_	3.3	τ_sg_	1.0
θ_*m*_	−37.0	*o*_*m*_	10.0
θ_*h*_	−58.0	*o*_*h*_	−12.0
θ_*n*_	−50.0	*o*_*n*_	14.0
θ_*r*_	−70.0	*o*_*r*_	−2.0
θ_*a*_	−57.0	*o*_*a*_	2.0
θ_*s*_	−35.0	*o*_*s*_	2.0
μ_*h*_	−40.0	γ_*h*_	−12.0
μ_*n*_	−40.0	γ_*n*_	−12.0
*k*_1_	30.0	*k*_Ca_	20.0
Γ	0.1 $\frac{\text{mol}}{\left({\text{lAs}}^{2}\right)}$	*c*_m_	1 $\frac{\text{pF}}{\mu m^{2}}$

The maximum conductances *g*_ion_ and reversal potentials *v*_ion_ were Gaussian distributed with a standard deviation of 5% around the respective mean values given in this table.

### 2.2. Three-dimensional model of the STN and the GPe

**Figure 2:**
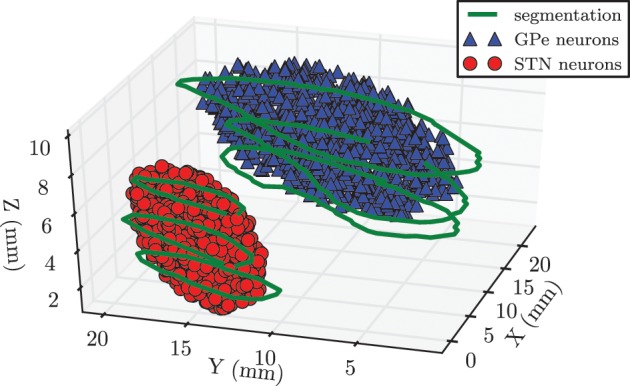
**Spatial model of STN and GPe being part of the left brain hemisphere**. Contours (shown in green) were segmented on MRI slices taken from a PD patient prior to DBS surgery. We approximated the spatial structure of the STN and the GPe with fits of two ellipsoids. Each of these volumes was filled with 10^4^ randomly distributed pointlike neurons.

In our network we modeled 10^4^ neurons each within the STN and the GPe. We had to make a trade-off between a physiologically realistic number of neurons in our system and the required computation time. Because the GPe fills a larger volume and has a lower density of neurons than the STN, it was reasonable to choose the same number of neurons for both nuclei (Levesque and Parent, 2005). In order to incorporate anatomical constraints conceding the spatial location of the STN and the GPe into our model, we made use of magnetic resonance imaging (MRI) data taken from a PD patient before DBS surgery. Both nuclei of the left brain hemisphere were segmented on slices of the MRI data by an experienced neurosurgeon. The segmentation contours are illustrated in green in Figure 2. In a second step we manually fitted an ellipsoid [given by (*x*/*a*)^2^ + (*y*/*b*)^2^ + (*z*/*c*)^2^ − 1 ≤ 0] into each of the obtained segmentation contours in order to approximate the anatomical shape of both structures. These two ellipsoid structures were filled with uniformly randomly distributed pointlike neurons. In this way we created a spatial model of the STN and the GPe, which is illustrated in Figure 2. The approximated values of the semi-principal axes for the two ellipsoids were: *a*_stn_ = 2.5 mm, *b*_stn_ = 6.0 mm, *c*_stn_ = 3.0 mm for the STN, and *a*_gpe_ = 4.6 mm, *b*_gpe_ = 12.3 mm, *c*_gpe_ = 3.2 mm for the GPe. Therefore, the resulting STN volume was *V*_stn_ = 188.5 mm^3^, which is in good accordance to a mean volume of 174.5 ± 20.4 mm^3^ reported in the literature (Levesque and Parent, 2005). The GPe volume is not presented here, because it is not the stimulation target and, thus, in the framework of our simulations the geometric dimensions are not crucial.

**Figure 3:**
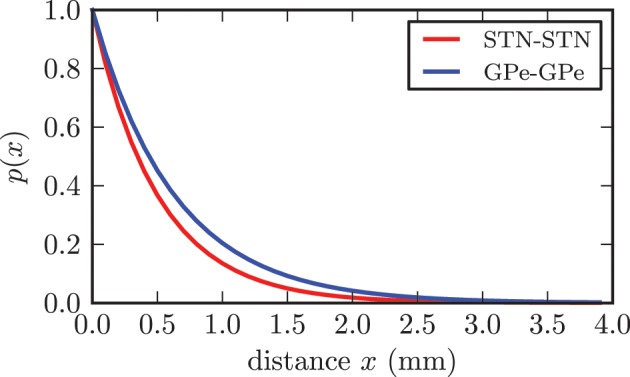
**Illustration of the dependence between connection probability and distance between two neurons**. Both functions fulfilled the constraint *p*(*r*_d_) = 0.33.

Although it is still up for discussion whether synaptic connections between STN neurons exist, there has been substantial experimental and theoretical evidence, which supports our assumption of the existence of excitatory connections among STN neurons (Gillies and Willshaw, 1998, 2004; Gillies et al., 2002; Shen et al., 2003; Shen and Johnson, 2006; Baufreton and Bevan, 2008). We based our connectivity value on a theoretical estimation given in Gillies and Willshaw (1998). Hence, each STN neuron extended connections to *p*_ss_ = 7% of the entire STN population, resulting in 700 synapses per neuron and 7 · 10^6^ synapses in total. The synaptic delays for all connections within the STN were extrapolated from values for gg connections reported in Holgado et al. (2010) and set to a homogeneous value of δ_ss_ = 4.0 ms. According to Holgado et al. (2010) we assumed that the corresponding time delays within both structures are shorter than the delays between GPe and STN. The connection probability between STN neurons declined exponentially with increasing distance (see Figure 3) and was given by *p*(*x*) = *e*^−*x*/*c*_*d*_^. The basis for our proposed distance dependence was the extent of the dendritic tree surrounding a STN neuron. In accordance with (Hellwig, 2000) we assumed that a synaptic contact is present when an axonal and a dendritic branch are spatially close to each other. Because the density of dendritic branches is lower for larger distances from the soma, the probability of a touch (and thus a synapse) between a dendritic tree and an axon decreases with increasing distance (Hellwig, 2000). The range of the dendrites varies from 0.2 to 0.9 mm between individual STN neurons and has a mean of *r*_*d*_ = 0.543 mm (Kita et al., 1983; Afsharpour, 1985). We adjusted the decay constant *c*_*d*_ = 0.5 such that the connection probability for the mean range of the dendritic tree *r*_*d*_ was still 33% and the probability for connections above the theoretical maximum distance of 1.9 mm was considerably low. While we chose a distance dependent probability for connections between neurons, the initial synaptic weights of the connections were Gaussian distributed around a mean value *w*_ss_(0) with standard deviation σ_ss_ without any distance dependence.

In contrast to the STN, the connectivity within the GPe was significantly lower with each neuron contacting only 1% of the entire population (Sadek et al., 2007), resulting in 100 synapses per neuron and 10^6^ synapses in total for our model. Connections between GPe neurons were inhibitory. We chose the same distance dependence as reported above, but adjusted the parameter *c*_*d*_ = 0.63 in accordance to the increased reach of the dendritic tree, which ranged to a maximum of 1.5 mm with a mean of *r*_*d*_ = 0.7 mm (Yelnik et al., 1984; Sadek et al., 2007). For the synaptic delay we chose δ_ss_ = 4.0 ms as suggested in Holgado et al. (2010). Similar to the connections within the STN, the synaptic weights were Gaussian distributed with a mean value of *w*_gg_ = 0.25 · 10^−3^ and standard deviation σ_gg_ = 0.125 · 10^−3^.

Besides the connections within the two simulated parts of the basal ganglia, both structures are also tightly interconnected (Shink et al., 1996). First, the STN has an excitatory influence on the GPe and on the other hand the GPe exerts an inhibitory impact on the STN neurons. These long range connections are established by axons extending from the STN to the GPe and vice versa. Therefore, we did not use distance dependent connection probabilities for links between STN and GPe, but we relied only on the connectivity values of 2% reported in Baufreton et al. (2009). This resulted in a fixed number of 200 synapses per neuron, contacting randomly chosen targets in the distant nucleus, respectively. The transmission delay of δ_sg_ = δ_gs_ = 4.0 ms for connections between the STN and the GPe is published in Fujimoto and Kita (1993) and Kita et al. (2005). Likewise to the above described connections, the initial values for the synaptic weight were Gaussian distributed around the mean values *w*_sg_ = 6.0 · 10^−3^ and *w*_gs_ = 3.0 · 10^−3^ with respective standard deviations σ_sg_ = 0.3 · 10^−3^ and σ_gs_ = 0.15 · 10^−3^.

### 2.3. Synaptic currents

In our model each incoming spike triggers a postsynaptic current with the shape of an α-function (Dayan and Abbott, 2000; Gerstner and Kistler, 2002). The postsynaptic current generated by a spike at time *t*_*k*_ reads:

(10) $$\alpha (t)=\frac{t-t_{k}}{\tau_{\text{syn}}^{2}}e^{-(t-t_{k})/\tau_{\text{syn}}},\;t_{k}\leq t<t_{k+1}$$

This yields the following coupling term, which quantifies the total synaptic input current to a postsynaptic neuron *i* received from presynaptic neurons *j*:

(11) $$I_{\text{syn}}(t)=\sum_{j}W_{ij}\left(v_{i}(t)-v_{\text{syn}}\right)\alpha (t)$$

*W*_*ij*_ denotes the synaptic weight (coupling strength) between presynaptic neurons *j* and the postsynaptic neuron *i*. *v*_syn_ is the reversal potential, at which a given neurotransmitter causes no net current flow of ions through the ion channels associated with that particular neurotransmitter receptor. Therefore, *v*_syn_ depends on the types of connected neurons and on whether the connection is excitatory or inhibitory. In our model, there exist four types of synapses for ss, sg, gs, and gg connections. The exact values of *v*_syn_ and τ_syn_ for the four different types of connections within our model are given in Tables 1, 2.

#### 2.3.1. Noise inputs

The external inhibitory input from the striatum to the GPe is modeled by a constant negative input current of *I*_app_ = −7.0 pA applied to all GPe neurons and additional Poissonian spike trains with a frequency of *f*^gpe^_p_ = 40 Hz.

(12) $$I_{\text{noise}}=\sum_{j\;=\;0}^{P_{\lambda }(i)}w_{\text{noise}}\left(v_{i}(t)-v_{\text{noise}}\right)\alpha (t),$$

with the Poisson distribution *P*_λ_(*i*) and λ = *f*_p_δ with the observation (simulated) time interval of the Poisson distribution δ between subsequent time steps, which is equal to the time resolution of the simulation. Likewise, we mimick the excitatory connections from the cortex to the STN also by feeding Poissonian distributed spikes with a frequency of *f*^stn^_p_ = 20 Hz into each STN neuron. We choose *f*^gpe^_p_ = 2 · *f*^stn^_p_, so that the physiological mean firing rate in the GPe is approximately twice the mean firing rate of the STN, as reported from experiments (Steigerwald et al., 2008; Nishibayashi et al., 2011; Wichmann et al., 2011). We do not feed the same spike train to all neurons, but each neuron receives a unique Poissonian spike train. For both the STN and the GPe the connection strength for the noise input is *w*_noise_ = 0.2, the value of the time constants for the α-function is τ_noise_ = 1.0 ms, and the reversal potential is chosen to be equal to *v*_noise_ = 0 mV.

### 2.4. Synaptic plasticity

We consider the synaptic strength of connections between STN neurons to be dynamical variables. Therefore, we introduce a learning rule that included synaptic weights, which are dependent on the timing of the firing of pre- and postsynaptic STN neurons (Gerstner et al., 1996; Markram et al., 1997; Bi and Poo, 2001). Our hypothesis that STN connections are governed by STDP is based on the findings reported in Shen et al. (2003), where long-term depression (LTD) and potentiation (LTP) could be induced by stimulation in rat STN neurons.

**Figure 4:**
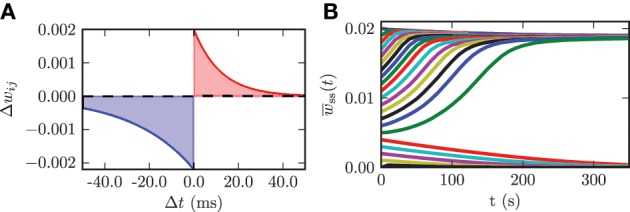
**(A)** STDP rule used for the connections among STN neurons in the model network. Depression is shown in blue and potentiation is shown in red. **(B)** Time evolution of the mean synaptic weight among STN neurons *w*_ss_(*t*) for different initial values *w*_ss_(0).

We use an additive spike timing-dependent plasticity (STDP) rule, which is illustrated in Figure 4A and defined by the following equation:

(13) $$\Delta w_{ij}\left(\Delta t\right)=\{\begin{array}{cc} \lambda e^{-\frac{\left|\Delta t\right|}{\tau_{\text{+}}}} & \;\Delta t>0,\\ -\lambda \beta e^{-\frac{\left|\Delta t\right|}{\tau_{-}}} & \;\Delta t\leq 0. \end{array}$$

In a previous study it has been shown that in a Kuramoto model of coupled phase oscillators multistability only occurs for asymmetric STDP rules (Maistrenko et al., 2007). This asymmetry was substantiated experimentally in different brain regions (e.g., hippocampus Bi and Poo, 1998 and neocortex Feldman, 2000). Therefore, we also used an asymmetric STDP rule with the following parameters: τ_−_ = 27.5, τ_+_ = 12.0, λ = 2.0 · 10^−3^, and β = 1.1. τ_−_ and τ_+_ are the time constants for the synaptic weight change of depression and potentiation, respectively. λ quantifies the impact of a single spike on the synaptic weight change and can be considered as the learning rate of the synaptic connection. The parameter β specifies the ratio between depression and potentiation in the synaptic learning rule. The synaptic weights among STN neurons are confined to the interval *w*^ss^_*ij*_(*t*) ∈ [0, *w*^ss^_max_], where *w*^ss^_max_ = 20 · 10^−3^. This upper boundary prohibits an unphysiological non-saturated increase in synaptic weights. We use hard bounds for the upper and lower boundary in our simulations. We use the same parameters for all synaptic connections among STN neurons. In order to investigate the dynamics of the model system with plasticity involved, we varied the initial mean synaptic weight among STN neurons *w*_ss_(0) and observed the time course of the mean synaptic weight *w*_ss_(*t*). The initial values for the mean coupling were Gaussian distributed with a standard deviation of 5% of the maximum weight σ_ss_ = 0.1 · 10^−3^. These simulations show that the system approaches one of the two dominating attractors with strongly synchronized activity (high mean coupling strength) or strongly desynchronized activity (low mean coupling strength). Below a certain threshold value in the initial mean synaptic weight *w*_ss_(0) = 5.0 · 10^−3^ the system monotonically approaches the desynchronized state, and with an initial value above the threshold the system monotonically converges to the strongly synchronized state (cf. Figure 4B). The plot shows only the time course of the mean synaptic weight among STN neurons within the first 350 s of neuronal activity. Nevertheless, we simulated the network activity for 600 s, in order to exclude transient or meta-stable behavior. Although STDP might induce many different multistable states, only the strongly desynchronized and strongly synchronized states predominate, and the system ultimately approaches either one of them. For instance, a strong initial connection strength among STN neurons leads to an inception of synchronized activity. This causes the neurons to fire nearly simultaneously, and this narrow spike timing results in a potentiation of the coupling strength and thus stabilizes the strong coupling and associated synchronization. In contrast, weak initial coupling is associated with desynchronized dynamics within the system and therefore results in a further decrease in synaptic connection strength. Hence, the desynchronized and synchronized dynamics are stable in the model system governed by plasticity. STDP facilitates the stabilization of synchronized and desynchronized activity and thus leads to multistability in the STN population. This observation is in good accordance with results from previous studies (Tass and Majtanik, 2006; Hauptmann and Tass, 2007; Popovych and Tass, 2012). The effects described above are robust against variations in the standard deviation of the initial mean coupling strength of up to 20% of the maximum weight.

#### 2.4.1. Stimulation input

For DBS the electrical stimulation is administered to the neuronal tissue in form of short biphasic current or voltage pulses (Cogan, 2008). A typical current pulse *P*(*t*) for stimulation of neuronal tissue has a cathodal and an anodal phase with current amplitudes and durations that result in an overall zero net charge for the entire pulse (to guarantee charge-balance):

(14) $$P(t)=\left\{ \begin{array}{l} \;\kappa \;t_{k}\leq t<t_{k}+\omega \;\\ -\kappa /p_{s}\;t_{k}+\omega \leq t<t_{k}+\omega (1+p_{s})\\ \;0\text{ else} \end{array}\right.$$

where *t*_*k*_ are the onset times of the current pulses, κ is the amplitude, and ω is the width of the cathodal pulse. *p*_*s*_ determines the duration and amplitude of the charge-balancing anodal pulse part. This charge-balance is important in order to avoid permanent charge transfer into the neuronal tissue, which might cause permanent tissue damage. For details of the possible electrochemical processes occurring at the electrode-tissue interface we refer to Cogan (2008).

The spatial arrangement of the stimulated neurons and the stimulation sites is crucial if the impact of electrical stimulation on a neuronal population is investigated. The influence of electrical stimulation on an individual neuron strongly depends on the relative positions within the three-dimensional coordinate system. One of the clinically most commonly used electrodes for DBS is the *Medtronic DBS lead model 3389*. The distal end with four electrode contacts is inserted into the target area. The electrode consists of four separate cylindrical contacts made of a Pt-Ir alloy with a typical length of 1.5 mm (Coffey, 2009). The electric field of such a cylindrical electrode is approximately radial and decreases inversely proportional to the distance. We used an analytic approximation based on the electric field of a line charge of finite length in order to estimate the distance dependence of the stimulation strength (Richardson et al., 2003). Furthermore, we assumed a homogeneous resistivity of 1.25 Ωm for the surrounding neuronal tissue. The approximation reads (Richardson et al., 2003):

(15) $$S\left(d_{ij}\right)=\frac{1}{d_{ij}l_{c}\sqrt{1+4{\left(d_{ij}/l_{c}\right)}^{2}}}$$

The function *S*(*d*_*ij*_) models the distance dependent decay of stimulation strength with *d*_*ij*_ representing the distance between neuron *i* and the location of the stimulation contact *j*. Additionally, *S*(*d*_*ij*_) depends on the length of the electrode contacts *l*_*c*_.

In order to eliminate the singularity at *d*_*ij*_ = 0, we assume a minimal distance of *d*_min_ = 0.7 mm to the electrode for all neurons. We achieve this by prohibiting possible neuron coordinates within a cylindrical volume with 1.4 mm diameter surrounding the electrode axis. This electrode canal is slightly larger than the electrode diameter and is created during the implantation surgery.

**Figure 5:**
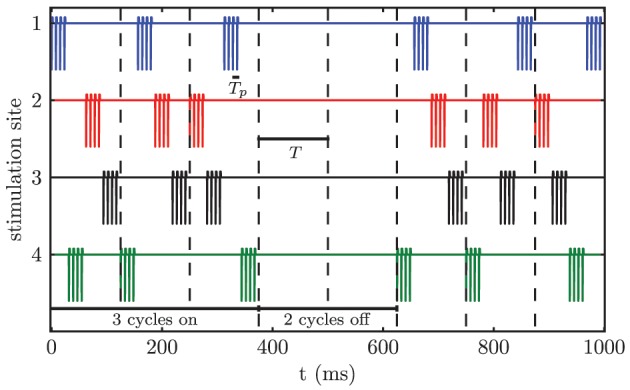
**Randomized CR stimulation signal**. The sequence of activated contacts within one cycle is randomized between cycles. The stimulation pattern consists of 3 ON-cycles and 2 OFF-cycles.

We apply CR stimulation (Tass, 2003a,c) to the modeled network of STN neurons. Each neuron receives stimulation input from all *M* stimulation contacts. The distance dependence of the stimulation strength causes the formation of *M* different sub-populations, because only the neurons close to a particular stimulation site are influenced sufficiently in order to alter their firing behavior. According to the CR stimulation protocol *M* different sub-populations are stimulated via *M* different stimulation sites at equidistant timing points by application of short bursts of current pulses within a stimulation period *T*, which approximately matches the period of the targeted pathological oscillation. *T*_*p*_ denotes the stimulation period within a burst. For our simulations we chose *M* = 4, which matches the number of available electrode contacts for the considered electrode type *Medtronic 3389*. In contrast to the sequential CR algorithm, we randomized the order of activated contacts between stimulation periods (i.e., stimulation cycles) (Figure 5). The only constraint was that the same contact could not be activated twice in a row between subsequent cycles. This modification of the CR stimulation protocol has previously been successfully used in an animal experiment with Parkinsonian monkeys (Tass et al., 2012b). Three cycles of CR stimulation (ON-cycles) were followed by two cycles without stimulation (OFF-cycles). According to a previous computational study this alternating pattern of 3 stimulation ON- and 2 stimulation OFF-cycles is superior to permanent CR DBS (without OFF-cycles), since during the OFF-cycles the CR-induced desynchronization is maximal (Lysyansky et al., 2011). Throughout our simulations we only varied the stimulation amplitude κ. The remaining stimulation parameters were fixed to *T*_*p*_ = 7.69 ms, ω = 200 μs, *p*_*s*_ = 8, and *T* = 125.0 ms.

Therefore, the effective stimulation signal received by neuron *i* from stimulation contact *j* is given by multiplication of the stimulation amplitude with the spatial profile defined by Equation 15 and reads:

(16) $$I_{\text{stim}}(t)=\sum_{j\;=\;1}^{M}S\left(d_{ij}\right)\rho_{j}(t)P(t)$$

The function ρ_*j*_(*t*) is an indicator function, which controls the activation of the respective stimulation site *j*. *M* is the number of activated stimulation contacts, where *M* = 4 for a typical electrode.

In our model we inject the stimulation current exclusively to the soma of the stimulated cells. Thus, our model omits various proposed effects of DBS (e.g., axonal activation). However, a recent computational study showed, that DBS of local STN cells located within the nucleus is more beneficial than the stimulation of fibers of passage (So et al., 2012). Nevertheless, our investigations revealed that the observed mixture of firing rates within the STN population is consistent with the interplay of effects reported or predicted for HF stimulation (Grill and McIntyre, 2001; McIntyre et al., 2004a; Miocinovic et al., 2006; Hauptmann and Tass, 2007). While some STN neurons showed stimulus-locked firing, mimicking the stimulus-locked somatic or axonal generation of action potentials in STN neurons, other STN neurons were completely or partially inhibited. Similar to Humphries and Gurney (2012) this mixture entirely arose from the interaction between the stimulation pulses fed into STN neurons and the effects induced by the reciprocal connections within the simulated STN-GPe network. This effect has also been reported previously for other simplified models of electrical stimulation (Humphries and Gurney, 2012). However, it is unquestioned that the mechanism of action of DBS includes suppression of synaptic input and direct (including retrograde) activation of axons (Ranck, 1975; Grill and McIntyre, 2001; McIntyre et al., 2004a; Miocinovic et al., 2006; Hauptmann and Tass, 2007; Gradinaru et al., 2009).

### 2.5. Simulation observables

Previous studies (Kuramoto, 1984; Daido, 1992; Tass, 1999) have shown that the phenomenon of synchronization within a neuronal model system can be quantified by the order parameter *R*(*t*). The order parameter *R*(*t*) bases on the phases ϕ_*j*_(*t*) of the individual neurons of the neuronal model system and is defined by:

(17) $$R\left(t\right)e^{i\psi \left(t\right)}=\frac{1}{n}\sum_{j\;=\;1}^{n}e^{i\phi_{j}(t)}$$

*n* denotes the number of neurons contained in the system. ψ(*t*) is the mean phase of the neuronal system and the quantity ϕ_*j*_(*t*) represents the phase of each individual neuron *j*, which is defined by linear interpolation between two subsequent spikes at times *t*_*j,k*_ and *t*_*j,k*+1_ (*k* = 0, 1, 2, …). Thus, the phase of a neuron ranges in the interval [0, 2π) and is given by the following equation (Pinsky and Rinzel, 1995):

(18) $$\phi_{j}\left(t\right)=\frac{2\pi \left(t-t_{j,k}\right)}{t_{j,k+1}-t_{j,k}},\;t_{j,k}\leq t<t_{j,k+1}$$

The above definition of a neuronal phase requires an adequate spike detection. We detected spikes by a combined threshold and local maximum search: If there was a local maximum above a certain threshold of the membrane potential, it was considered a spike. We performed the spike detection with an accuracy of 0.1 ms, which was sufficient according to a typical spike width at half-maximal spike amplitude of approximately 2.0 ms. We did not distinguish between single spikes and bursts (episodes of rapid spiking). *R*(*t*) quantifies the coherence of the phases and is ranging from 0 to 1, where *R*(*t*) = 1 corresponds to a fully synchronized system, while values close to zero correspond to complete absence of phase synchronization. Due to finite size fluctuations *R*(*t*) never reaches zero, $\frac{1}{\sqrt{n}}$ is the expected value. We measured the degree of synchronization separately for the STN and GPe sub-networks. For example, in the desynchronized regime the time course of the first order parameter *R*(*t*) is stable and close to zero in both neuronal populations with time averages of 〈*R*〉_stn_ = 0.009 and 〈*R*〉_gpe_ = 0.009, which are in good accordance with the expected value of *R*(*t*) = $\frac{1}{\sqrt{n}}$ = 0.01. In contrast, in the synchronized regime *R*(*t*) is stable and close to 1 for the STN sub-network with an average value of 〈*R*_1_〉_stn_ = 0.97 indicating almost perfect in-phase synchronization. For the GPe population the values of the order parameter are considerably lower with a time average of 〈*R*_1_〉_gpe_ = 0.67. The weakly coupled GPe neurons are periodically synchronized by the external force of the STN. In order to smooth out short-term fluctuations and highlight long-term trends, we calculated a moving average over the recorded data. Thereby we used a time window of 1000 ms duration.

In order to predict an optimal location for the DBS electrode we additionally studied the spatial distribution of synchronization within the STN volume. Therefore, we introduced a method to measure local synchronization within the STN, based on the order parameter described above. In our approach we subdivided the volume of the STN in equally sized cubic voxels and computed the order parameter *R*_*k*_(*t*) for the set of *N*_*k*_ neurons contained within each voxel *k* separately.

(19) $$R_{k}\left(t\right)e^{i\psi_{k}\left(t\right)}=\frac{1}{N_{k}}\sum_{j\;=\;1}^{N_{k}}e^{i\phi_{j}\left(t\right)}$$

Synchrony was calculated for each voxel individually. The voxels do not overlap. The high density of neurons in our large-scale model guarantees that each voxel contains approximately *N*_*k*_ ≈ 42 neurons. Nevertheless, we only considered voxels containing at least 10 neurons. For voxels with a low number of contained neurons the distinction between partly and fully synchronized states can become difficult due to the finite size fluctuations mentioned above. This also limits the spatial resolution of this synchrony measure. In order to estimate the homogeneity of local synchronization within the STN volume, we define the following local order parameter:

(20) $${\bar{r}}_{1}=\frac{1}{N_{\text{V}}}\sum_{k\;=\;1}^{N_{\text{V}}}\langle R_{k}\rangle$$

*r*_1_ is close to one if the entire STN volume is strongly synchronized and close to zero if it is strongly desynchronized. *N*_V_ = 232 denotes the number of voxels considered for the calculation. Note, *r*_1_ is close to one if either the whole STN population is synchronized and constitutes a giant oscillator, or if the STN population is synchronized on the level of single voxels only, whereas between different voxels there might be non-vanishing phase shifts. The standard deviation of this local order parameter σ_*r*_1__ incorporated additional information about the homogeneity of the local synchronization. Large values of σ_*r*_1__ indicate high discrepancies in the local order parameter between different regions of the STN volume.

## 3. Results

### 3.1. Scalability test

**Figure 6:**
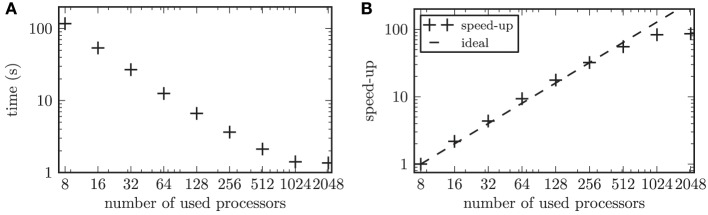
**Results of the scalability test. (A)** shows the time to simulate 1 biological second of the network activity as a function of the number of used processors in double logarithmic representation. **(B)** displays the speed-up factor for the simulation times. The symbols (+) represent the measured speedup, while the dashed line marks the ideal speed-up.

The large number of simulated neurons made it necessary to parallelize the numerical computations. In our performance tests, we focused on the strong scaling of our model. Strong scaling characterizes the ability of an application to perform the same sized calculation faster on computers with more processors (Plesser et al., 2007). The network was built according to the scheme described above. Thus, the resulting network contained 2 · 10^4^ nodes connected via 1.2 · 10^7^ synapses. Only the synaptic weights between the STN neurons were governed by a STDP rule, while all remaining connections were constant in time. We focused on the simulation of the network activity and omitted the online data analysis in the scalability tests. The time evolution of this network was simulated for one biological second and the runtime was measured. To test the strong scaling behavior of the simulation program, the problem size was fixed and the number of used processors on the supercomputer was increased from 8 to 2048 processors. During simulations the neurons of the entire simulated network were active with a mean firing rate of 15.8 Hz. The scalability of our simulation is illustrated in Figure 6. The speed-up factor quantifies how much faster the simulation runs on *n* processors, with runtime *T*_*n*_, compared to a simulation on 8 processors, (*T*_8_). Thus, the speed-up factor is given by *S* = *T*_8_/*T*_*n*_. For up to 128 used processors our scalability test showed a slightly steeper slope than the ideal linear speed-up (see Figure 6B, i.e., for up to 128 processors the speed-up marks “+” are positioned above the ideal line of linear speed-up). A maximal slope for the speed-up of 1.1 was observed (averaged over 10 independent trials). Although the linear speed-up is commonly accepted to be the theoretical maximum, this supralinear behavior of our simulation can be explained by the fast cache memory increasing proportionally to the number of used processors (Morrison et al., 2005). Thus, the ratio between the locally required working memory and the locally available cache memory decreases, which leads to a performance gain (Wilkinson and Allen, 2004). Note, the supralinear speed-up might be more prominent for simulated networks with higher average firing rates (Morrison et al., 2005). This is because the size of the available cache memory has a stronger impact on the efficiency of writing to the spike buffers for higher firing rates of the simulated neurons.

The computational workload of the individual neurons in a simulated neuronal network has a significant impact on the scaling behavior of the simulation (Plesser et al., 2007). This is due to the fact that a computationally expensive model for individual neurons or a larger number of neurons per processor leads to an increase in the ratio of local computation to communication costs. The overhead due to the communication in a distributed simulation grows with an increasing number of used processors (Plesser et al., 2007). In our performance test this effect balanced the performance gain from efficient caching for 256 processors. The communication overhead became predominant for 2048 processors, which led to a saturation of the gained speed-up. In the simulations we performed throughout this work, we typically used 1024 processors. With this setup the simulation of one biological second consumed approximately 3 s of computation time. This included the required computation time for the online analysis of the simulation results, which was omitted in the scalability tests.

#### 3.1.1. Spontaneous activity of the model network

As shown above the system enters one of the two dominant stables states depending on the initial mean synaptic weight among the STN neurons. The observed spontaneous activity within the two states can be characterized in the following way: First, for low mean coupling within the STN the neurons fire in an uncorrelated, desynchronized manner. We consider this type of activity as a model for the non-pathological (healthy) state of the basal ganglia system. Second, the system is capable to fire in a strongly correlated synchronized pattern, if the mean coupling strength within the STN is strong enough. This type of activity models the pathological (“Parkinsonian”) state of the STN-GPe network.

Both states can be characterized by multiple observables, e.g., the mean membrane potentials of both populations that have been recorded with 1.0 ms time resolution. In the desynchronized state the mean membrane potentials of the STN and the GPe population showed only minor fluctuations around the resting value. The time averages over the mean membrane potentials were 〈*v*(*t*)〉_stn_ = −59.2 ± 0.1 mV and 〈*v*(*t*)〉_gpe_ = −90.2 ± 0.1 mV.

In order to measure the degree of synchronization within the simulated neuronal network, we therefore calculated the order parameter *R*(*t*) (section 2.5), which measured the phase coherence of the neurons and gave us the opportunity to detect possible cluster states. The time course of the order parameter *R*(*t*) was stable and close to zero in both neuronal populations with a time average of 〈*R*(*t*)〉_stn_ = 0.009 for the STN and 〈*R*(*t*)〉_gpe_ = 0.009 for the GPe with standard deviations smaller than 10^−4^. The time course of the order parameter for both sub-structures was in accordance to what could be expected from a system of noise driven and very weakly coupled oscillators.

**Figure 7:**
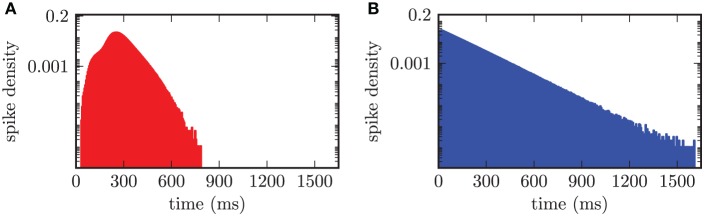
**ISI histogram (semi-logarithmic plot) for both neuronal populations in the desynchronized state. (A)** Slightly asymmetrical distribution of ISIs for the STN population with a median of $\tilde{T}$_stn_ = 261.2 ms. **(B)** Obvious asymmetry in the ISI histogram, due to bursting activity of the GPe neurons, with $\tilde{T}$_gpe_ = 92.1 ms.

An adequate characterization of the observed neuronal activity required the investigation of the firing frequencies of individual neurons and their distribution. Therefore, we calculated the time intervals between subsequent spikes for each neuron separately and thus obtained the distribution of interspike intervals over the two populations of neurons. Hence, this resulted in an interspike interval (ISI) histogram shown in Figure 7. The bin width of the histogram was chosen equal to 5.0 ms. In the desynchronized state the ISI histogram of the STN population showed a broadened distribution of ISIs around the median value of $\tilde{T}$_stn_ = 261.2 ms with an average absolute deviation from the median (MD) of MD_stn_ = 51.1 ms. Accordingly, this value corresponded to a median firing rate of $\tilde{f}$_stn_ = 3.8 Hz. For the GPe neurons we calculated a median spike period of $\tilde{T}$_gpe_ = 92.1 ± 82.6 ms, which corresponded to a median spike frequency of $\tilde{f}$_gpe_ = 10.9 Hz. The ISI distribution for the GPe was asymmetrical and is shown in Figure 7B. The asymmetry occurred due to the fact that the GPe neurons have a tendency to fire short bursts of action potentials. These bursts had relatively short ISIs resulting in a shift of the histogram to ISIs with reduced length. We preferred calculating the median over the mean value, because both distributions were asymmetrical and for a symmetrical distribution mean and median have identical values.

For strong mean coupling among STN neurons (*w*_ss_ > 5.0 · 10^−3^) the model system showed highly synchronized activity (*R*(*t*) ≈ 1). The synchronized state can be considered as the pathological, unhealthy state, which mimics the neuronal activity within the basal ganglia system in patients suffering from PD (Lenz et al., 1994; Plenz and Kital, 1999). The STN neurons fired collectively with a median period of $\tilde{T}$_stn_ = 122.0 ± 8.3 ms, which corresponded to a median firing rate of $\tilde{f}$ = 8.2 Hz. Between these episodes of collective firing the individual STN neurons remained almost silent. The GPe neurons were driven by the STN activity and showed collective firing with the same frequency as the STN neurons. Nevertheless, individual GPe neurons emitted action potentials constantly and collective periods of silence were not observed for the GPe population.

**Figure 8:**
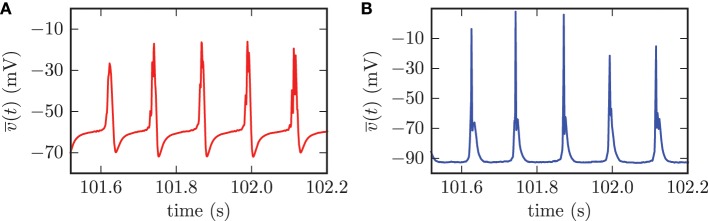
**For the synchronized state, the mean membrane potentials of the neurons within the STN (A) and the GPe (B) showed oscillations with large amplitudes**. The frequency of these oscillations was the same for both populations.

The computation of population mean membrane potentials gives similar results. The mean membrane potentials of both populations showed high amplitude oscillations with the same frequency as the synchronized activity of the STN neurons (cf. Figure 8). The synchronized firing of the STN neurons forced periodic collective spike discharges of the GPe neurons. This collective GPe activity led to an increased inhibition of the connected STN cells. Thus, the mean membrane potential of the STN population was strongly hyperpolarized (cf. Figure 8A). This mechanism was the reason for the periods of silence in STN neurons following the episodes of collective firing. However, the inhibition from GPe neurons decayed and the mean membrane potential of the STN population recovered before the next episode of collective firing was launched. The GPe neurons, which were excited by incoming action potentials from collective firing of the STN neurons, reacted to this excitation with the emission of bursts. This bursting activity in the GPe neurons caused the very narrow peaks in the mean membrane potential of the GPe population (cf. Figure 8B).

A very clear indication of synchronized activity in the simulated network was furthermore provided by the time course of the order parameter *R*(*t*). For the STN sub-network *R*(*t*) was stable and close to 1 with an average value of 〈*R*(*t*)〉_stn_ = 0.97 indicating almost perfect in-phase synchronization. However, perfect in-phase synchronization was never achieved, because the STN sub-network was composed of non-identical individual neurons and the system always received noise inputs from surrounding structures. For the GPe population the values of the order parameter were considerably lower oscillating around a time average of 〈*R*(*t*)〉_gpe_ = 0.67. This finding can be explained by the fact that the weakly coupled GPe neurons fired in a desynchronized manner most of the time and are periodically synchronized by the external force of the STN. The synchronized input from the STN triggered the collective bursting of the GPe neurons and afterwards the GPe population returned to the desynchronized firing pattern.

**Figure 9:**
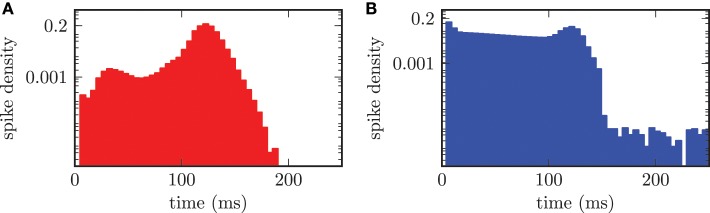
**ISI histogram (semi-logarithmic plot) for both neuronal populations in the synchronized state. (A)** The STN neurons synchronize with a median period of $\tilde{T}$_stn_ = 122.0 ms. **(B)** Two maxima in the ISI histogram of the GPe population due to bursting activity of the GPe neurons and the external forcing from the STN.

The effect of synchronization on the distribution of ISIs is clearly visible in Figure 9. In the synchronized state the median period of spiking activity in STN neurons was shifted toward shorter durations $\tilde{T}$_stn_ = 122.0 ± 8.3 ms. The consequence was an increased frequency $\tilde{f}$_stn_ = 8.2 Hz and much sharper distribution of ISIs (cf. Figure 9A), than for the desynchronized network activity, shown in Figure 7A. The calculation of the median or mean did not make sense for the bi-modal distribution of ISIs in the GPe population shown in Figure 9B. The bi-modal distribution was caused by two reasons. First, the large peak for short time periods around 5 − 10 ms could be explained by the increased bursting activity of the GPe neurons. Second, the clearly visible peak at approximately 122.0 ms was caused by the driving force of connected STN neurons. Additionally, the entire ISI histogram for the GPe population indicated an increased activity distributed over all time periods. This was due to the fact that the frequency of the input from the STN to the GPe was increased in the synchronized regime.

### 3.2. Optimal stimulation amplitude

**Figure 10:**
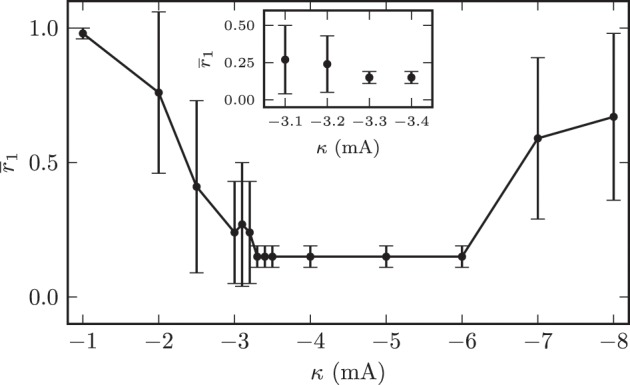
**Dependence of the local synchronization parameter *r*_1_ measured 420 s after cessation of stimulation on the used stimulation current amplitude**. The plot demonstrates an amplitude range associated with a complete desynchronization of the STN volume.

In all subsequent simulations we set the initial mean synaptic weight among STN neurons to *w*_ss_ = (18.0 ± 0.1) · 10^−3^. This corresponded to an initially strongly synchronized activity of the model system. In the first 120 s of the simulations we left the system unperturbed before we switched on the CR stimulation. As described above, we applied the randomized CR stimulation sequences (Figure 5). The contacts of the stimulation electrode were placed along the *y*-axis of the coordinate system. The *M* = 4 electrode contacts were centered within the STN volume with a distance of 2 mm between two neighboring stimulation sites. In our simulations we only varied the amplitude of the stimulation current pulses κ and the location of the electrode contacts. The remaining stimulation parameters were fixed and given by: ω = 200.0 μs, *p*_*s*_ = 8.0, *T*_*p*_ = 7.69 ms, *M* = 4, *T* = 125.0 ms, and number of OFF- and ON-cycles= 3 and 2. The first question we addressed was to look for an “optimal” stimulation amplitude. An optimal stimulation amplitude results in sufficiently strong effects, i.e., desynchronization of the target structure, while using a minimum stimulation amplitude to avoid potential side effects. Therefore, we increased the applied stimulation amplitude in a stepwise manner and investigated the desychronizing effect of the respective stimulation current amplitude κ. We performed this investigation for fixed electrode contact locations in the center of the STN volume. The value of the local order parameter *r*_1_ obtained after stimulation application is used as a quantitative measure for the desynchronization capabilities of a particular stimulation amplitude. The order parameter was averaged in time (interval *t* ∈ [1140, 1200] s, i.e., the last 60 s of simulated neuronal activity). The averaging started 420 s after cessation of stimulation. Figure 10 illustrates the dependence of the local order parameter *r*_1_ on the stimulation amplitude κ. The illustration shows that for low stimulation amplitudes a significant decrease in synchronized activity could not be achieved. For instance, with an amplitude of κ = −1.0 mA the system remained in a strongly synchronized state after stimulation, and the low standard deviation for *r*_1_ indicated that the entire STN volume was homogeneously synchronized. This was because for these low amplitudes the stimulation signal was not strong enough, and neurons in further distance from the electrode contacts did not sense a sufficiently strong input current in order to achieve a reliable phase reset. Hence, the amount of neurons recruited by stimulation was not large enough for very low stimulation amplitudes.

**Figure 11:**
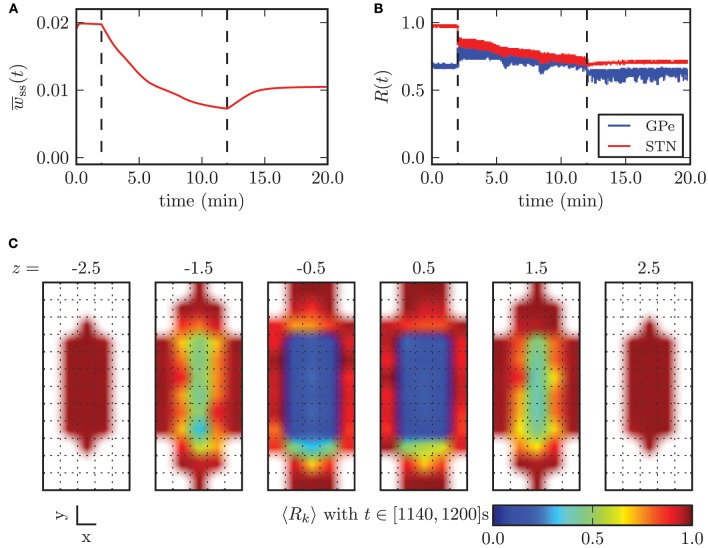
**Outcome of CR stimulation with insufficient amplitude (κ = −2.0 mA)**. The dashed lines indicate when the stimulation was switched on/off. **(A)** The mean synaptic weight decreased during stimulation, but this effect did not persist after cessation of stimulation, after which the mean weight slightly recovered. **(B)** The order parameter within the STN population was reduced, while it stayed at the same level in the GPe, but with larger fluctuations. **(C)** shows that desynchronized neuronal activity was restored only within the inner core of the STN volume.

With further increasing amplitude, the fraction of neurons sensing significant stimulation inputs linearly increased and the electrical CR stimulation unfolded its desynchronizing effect. Nevertheless, large standard deviations of *r*_1_ for stimulation amplitudes below −3.2 mA indicated the existence of large discrepancies in the spatial distribution of the local order parameters. For illustration, the effects of CR stimulation with amplitude κ = −2.0 mA on the model system are shown in Figure 11. The mean synaptic weight was successfully decreased during stimulation, but not sufficiently enough in order to shift the system into an overall strongly desynchronized state. After the stimulation the mean synaptic weight increased again and stabilized at *w*_ss_(*t* → ∞) ≈ 10.4 · 10^−3^. Accordingly, the order parameters were reduced, but both values after stimulation (〈*R*_1_〉_stn_ = 0.7, 〈*R*_1_〉_gpe_ = 0.63) indicated the persistence of reduced, but still highly correlated activity within the STN and GPe sub-networks. Figure 11C shows the spatial distribution of the local order parameter after CR stimulation and provides an explanation for these observations. Only the inner part of the STN volume was reliably desynchronized, while synaptic connections and thus synchronization among neurons at larger distances from the electrode could not be reduced due to an insufficient stimulation amplitude. However, these patterns of local (de-)synchronization after CR application were stable within our observation times. Above we showed that for spatially homogeneous initial conditions the system approaches one of the two dominating attractors with strongly synchronized activity (high mean coupling strength) or strongly desynchronized activity (low mean coupling strength) cf. Figure 4B. Our observations show that the coupling strength between neurons can be influenced locally by electrical CR stimulation and thus the system can be forced to approach one of the stable attractors shown in Figure 4B if the fraction of manipulated connections is large enough.

This figure also indicates that the synaptic connection strengths between STN neurons, which were governed by a STDP learning rule, converged either to values close to the maximum synaptic weight *w*^max^_ss_ = 20.0 · 10^−3^ or to values close to the minimum of *w*^min^_ss_ = 0. The minimal stimulation amplitude, which was capable to achieve a persistent desynchronization of the complete STN volume, was κ_opt_ = −3.3 mA. The stimulation outcome did not differ qualitatively for stimulation amplitudes above κ_opt_. However, the effective desynchronization of the STN volume broke down for amplitudes above −6.0 mA. This effect could be explained by a reduced spatial specificity of the stimulation for larger amplitudes, i.e., the regions stimulated by the different electrode contacts showed high intersections among each other, which led to stimulation of the entire STN population instead of small sub-populations of STN neurons.

For intermediate amplitudes (i.e., κ ∈ [−3.3 mA, −6.0 mA]) and during active CR stimulation the STN is divided into four synchronized sub-populations. The four synchronized sub-populations origin from the four stimulation sites, i.e., the periodic stimulation at each individual stimulation site causes a local synchronization of the surrounding neurons. Through the four stimulation sites a phase-shifted stimulation pattern (i.e., the typical CR pattern) is applied, which results in an overall weakening of synaptic weights and thus decreases the synchronized activity of the whole STN population already during stimulation.

For larger amplitudes almost all STN neurons are synchronized and form one population which is firing in phase. This synchronized firing does not decrease synaptic weights.

**Figure 12:**
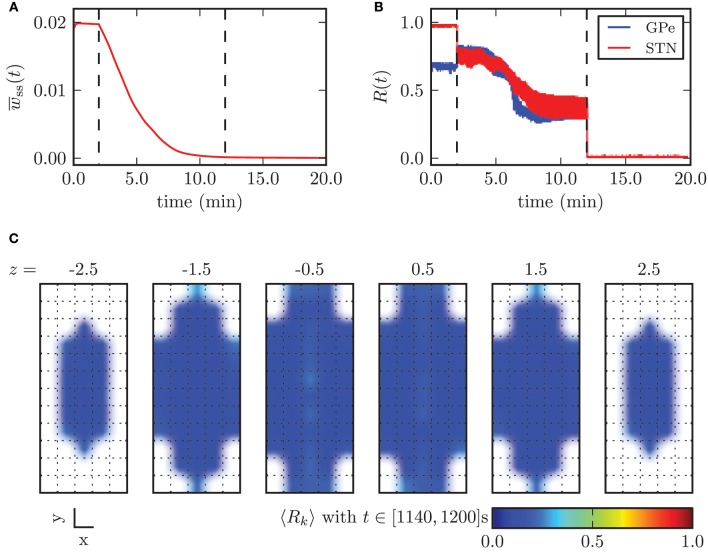
**Outcome of CR stimulation with appropriate amplitude (κ = −3.3 mA). (A)** Mean synaptic weight among STN neurons and **(B)** time course of the order parameter. The points in time when the stimulation was switched on and off are marked by the vertical dashed lines. **(C)** shows the spatial distribution of synchronization and illustrates the homogeneous desynchronization in the whole STN volume.

In Figure 12 we illustrated the effects of CR stimulation at appropriate intensity, by considering the results obtained with the minimal stimulation amplitude enabling complete desynchronization, κ_opt_ = −3.3 mA. Before the stimulation was switched on the mean synaptic weight among STN neurons was stable and close to its maximum value. During the application of CR stimulation the mean weight *w*_ss_ monotonically decayed. After 10 min the continuous CR stimulation was switched off and without further stimulation the mean synaptic weight stabilized close to zero (Figure 9A). The order parameters for the STN and the GPe population decreased during stimulation, but saturated and fluctuated around a value of approximately *R*_1_ = 0.35. This is due to the fact that during application of CR stimulation sub-populations of the STN are periodically synchronized before they undergo a desynchronization transient. Nevertheless, after switching stimulation off both neuronal populations fired in a strongly desynchronized manner and remained in this state.

### 3.3. Influence of electrode location

**Figure 13:**
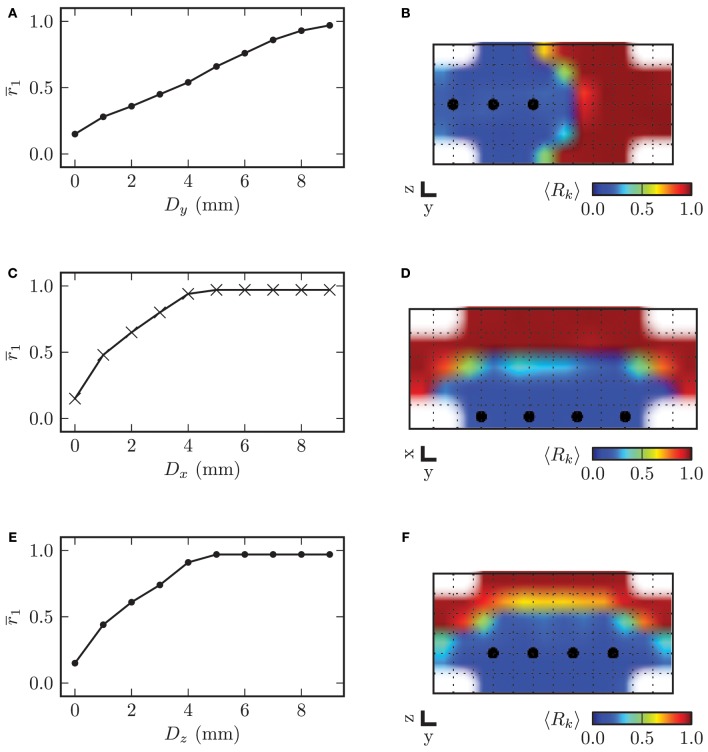
**On the left hand side the displacement of the electrode along the respective coordinate axis from the reference position in the center of the STN volume is plotted against the mean local order parameter *r*_1_ after stimulation**. The y-axis points into the direction of electrode implantation. On the right hand side the spatial distribution of the local order parameter is shown for specific values of *D*. **(A)** electrode displacement along *y*-axis vs. local order parameter **(B)** example for *D*_y_ = 4.0 mm **(C)** electrode displacement along *x*-axis vs. local order parameter **(D)** example for *D*_*x*_ = 2.0 mm **(E)** electrode displacement along *z*-axis vs. local order parameter **(F)** example for *D*_*z*_ = 1.0 mm. Plots **(B)** and **(F)** show a cross-section of the (*y*, *z*)-plane with *x* = 0 mm, and **(D)** displays a cross-section through the (*x*, *y*)-plane with *z* = −0.5, mm.

In order to investigate the influence of the stimulation electrode position within the STN volume on the desynchronizing effect of CR stimulation, we varied the electrode position, i.e., the location of the array of stimulation contacts and analyzed the stimulation outcome in comparison to an ideal reference position. The topology of the array of stimulation contacts remained constant. The electrode position with the four electrode contacts centered in the STN volume served as reference position. Starting from this reference position, we consecutively moved the electrode along the three respective coordinate axes out of the center of the target in steps of 1.0 mm. For all simulations we used the stimulation amplitude of κ = −3.3 mA and the same stimulation parameters as before. The mean local order parameter *r*_1_ was computed over the last 60 s of simulated neuronal activity *t* ∈ [1140, 1200] s and after cessation of stimulation. In Figure 13 we display the dependence of the local order parameter *r*_1_ after CR stimulation on the displacement *D* along the three respective coordinate axes from the reference position. With the electrode located at the reference position the desynchronization of the STN volume was most effective. With further distance from the reference position the local order parameter *r*_1_ increased monotonically for growing displacements of the electrode irrespective of the considered coordinate axis.

The dependence of *r*_1_ on the displacement along the *x*- and *z*-axis was very similar. This was caused by the fact that the ellipsoid defining the boundary of the STN volume was almost symmetrical in *x*- and *z*-direction with values for the semi-principal axes of *a*_stn_ = 2.5 mm and *c*_stn_ = 3.0 mm. The slope of the increase in *r*_1_ was significantly lower for the displacement along the *y*-axis, because the volume of neurons stimulated with a sufficiently high amplitude decreased with a lower rate than for displacement of the electrode along the two other coordinate axes. Therefore, we can conclude that the effect of electrode displacement on the stimulation outcome had a more significant impact for deviations along the *x*- and *z*-axes than along the *y*-axis.

However, as mentioned before, the increase in stimulation amplitude is limited by several factors. First, an effective desynchronization could not be achieved for amplitudes which exceeded a certain (model dependent) threshold, as showed above. Second, larger stimulation amplitudes might induce unwanted side effects via unintentional stimulation of other brain areas or fiber tracts (Smith et al., 2012). Third, high stimulation current amplitudes might induce permanent damage to the neuronal tissue surrounding the electrode (Grill and McIntyre, 2001; Grill, 2005; Cogan, 2008).

## 4. Discussion

In this study we developed a large-scale model of two basal ganglia structures STN and GPe, which are hypothesized to generate rhythmic synchronized oscillations. This synchronized activity is associated with symptoms arising in PD (Lenz et al., 1994; Plenz and Kital, 1999). The described model is the continuation of our top-down approach (Tass, 1999, 2003c; Popovych et al., 2005; Tass and Majtanik, 2006; Hauptmann and Tass, 2007, 2009, 2010; Tass and Hauptmann, 2009). By gradually increasing the complexity of our model, we were able to link our findings to previous results obtained from models with a reduced level of detail. Therefore, we increased the model size, parallelized the simulations and data analysis. Thus we were able to achieve a simulation performance close to real-time. We investigated and characterized the two predominant stable synchronized and desynchronized states, which modeled the pathological and healthy state of the basal ganglia system, respectively. We showed that the transition between both predominant states was dependent on the mean coupling strength among STN neurons. This observation is in good accordance with previous modeling studies (Hauptmann and Tass, 2007, 2009; Tass and Hauptmann, 2007, 2009). In addition, with our large-scale approach we were able to reveal clinically relevant spatio-temporal effects: spatially incomplete desynchronization caused by either insufficient stimulation or by electrode displacement.

Furthermore, we investigated the effects of CR stimulation on the network activity and, in particular, on the synaptic structure of the STN sub-network. In this context we were especially interested in how the neuronal network can be shifted from the strongly synchronized to the desynchronized mode of network activity. To this end, we introduced a synaptic learning rule and a measure enabling to assess the distribution of synchronized neuronal activity within the volume of the STN. In our investigations of the desynchronizing CR stimulation technique, we observed a certain amplitude range for which desynchronization of the target structure was most effective. This finding might be useful for further experimental studies. This optimal parameter range has been reported previously for simpler models of coupled phase oscillators, FitzHugh-Nagumo spiking neurons, FitzHugh-Rinzel bursting neurons, and Hodgkin-Huxley neurons with and without STDP (Guo and Rubin, 2011; Lysyansky et al., 2011; Popovych and Tass, 2012). Additionally, in an experimental study in parkinsonian non-human primates it was shown that CR DBS at low intensity has long-lasting after effects on basal ganglia motor function (Tass et al., 2012b) and a clinical proof-of-concept trial revealed that CR DBS has lasting aftereffects in patients with Parkinsons disease as assessed by UPDRS III scores and local field potential theta and beta activity (Adamchic et al., 2014a).

In this study we expanded these observations in a more complex and microanatomically more realistic model. We incorporated more realistic coupling topologies and took the spatial arrangement of the electrode and stimulated neurons into account. However, the observed amplitude parameters highly depend on how the electric field surrounding the electrode is modeled (Richardson et al., 2003; Butson et al., 2006; Chaturvedi et al., 2010; Schmidt and van Riemen, 2012). Therefore, a generalized quantitative prediction of optimal stimulation parameters is difficult to obtain from a theoretical simulation study. Additionally, the spatial structure enabled us to vary the electrode location within the stimulation target and thus we observed a strong relation between the electrode position and the stimulation outcome. This observation substantiates previous theoretical and experimental studies, which investigated the impact of the electrode position on the stimulation outcome for HF DBS (Voges et al., 2002; Paek et al., 2011; Guo et al., 2012; Nakano et al., 2012; Reese et al., 2012).

Because of the high computational costs of our complex model, we limited our observations of CR DBS effects to timescales of several 10 of minutes. Therefore, we used a STDP learning rule which showed adequate effects on such timescales. Our model could adequately reproduce the experimentally observed long-term desynchronization effects of CR stimulation, which last on timescales from minutes to hours and days, demonstrated in slice experiments (Tass et al., 2009), a pre-clinical proof-of-concept study (Tass et al., 2012b) and a proof-of-concept trial (Adamchic et al., 2014a). Certainly, the learning rule has a significant impact on the simulation results. Therefore, more experimental data specifying the actual plasticity mechanisms in the basal ganglia and especially within the STN are required. In order to be able to simulate the long-term effects (timescales of days or weeks) of CR DBS, a modification of the STDP rule has to be considered. However, these long-term simulations necessitate a further improvement of the simulation code in terms of scalability and performance or a reduction of the model complexity. At this point less complex models might be more suitable for the investigation of long-term DBS effects, but with steadily increasing performance and computing capacity these long-term simulations will be possible in the medium-term.

We considered many physiologically relevant parameters that have been neglected in former simplified models of the STN-GPe network (e.g., spatial extent, realistic neuronal connectivity, and dimensionful neuron model) (Hauptmann et al., 2005; Feng et al., 2007; Tass and Hauptmann, 2009; Guo and Rubin, 2011; Lysyansky et al., 2011). This complex modeling approach had the advantage that characteristics observed in the model activity might be more transferable to an experimental verification. However, there are still some limitations and simplifications in our model. We used a simplified approximation for the distance dependence of the electric field (and thus the stimulation strength) surrounding the electrode. Besides the assumption that the electric field strength behaves similar to the radial component of the field for a finite line charge, this approximation was based on a homogeneous and isotropic tissue conductance. In a future refinement of the presented model the implementation of more realistic representations of the electric field (e.g., based on finite element methods McIntyre et al., 2004b) is intended (Butson and McIntyre, 2005, 2006; Butson et al., 2006; Chaturvedi et al., 2010; Schmidt and van Riemen, 2012). However, if a “realistic" representation is desired it is also necessary to consider the movements of charge carriers in the surrounding tissue (e.g., extracellular fluid). Obviously, substantiation of such a modeling approach requires more experimental data. Also, in this study we trade-off model complexity, to reduce computational costs. Accordingly, we introduced STDP only within the STN, since this is the primary stimulation target for CR DBS.

By the same token, future studies might incorporate more complex STDP rules including soft bounds (van Rossum et al., 2000). The effects of hard vs. soft bounds in a similar system were studied in detail in Popovych et al. (2013). That study provides a systematical investigation of the effects of hard and soft bounds on spontaneous dynamical states (in particular, multistability) as well as stimulation-driven dynamical states (stochastic resonance induced by uncorrelated noise) of a network. The stochastic resonance was robust with respect to these modifications. Furthermore, multistability, an essential ingredient for CR to induce long-lasting effects, as well as robust anti-kindling was observed for hard bounds (Tass and Majtanik, 2006) as well as for soft bounds (Maistrenko et al., 2007) in phase oscillator networks. Correspondingly, to demonstrate that the CR-induced anti-kindling does not depend on the particular choice of bounds in the model used in this paper, among other future developments we are planning to take into account soft bounds in a forthcoming study, too. Moreover, further studies might be used to investigate more complex phenomena in the field of STDP, for example the observation that in the presence of propagation delays in recurrent networks exhibiting population bursts, STPD rules might exert a strong decoupling force which can further the desynchronization of the network activity (Lubenov and Siapas, 2008). Also, STDP can induce oscillatory behaviors depending also on the model parameters and in particular on the shape of the considered pulse (Mikkelsen et al., 2014). Further studies might also incorporate neighboring structures which are omitted in the current approach. For example, GPe fibers and axonal pathways, which might be relevant for a detailed analysis of the effects of variations of the electrode position (Rubin et al., 2012). Additional measures, focussing on the effect of information transfer on antidromic structures might further increase the understanding of the therapeutic effect of different sites and types of stimulation (Rubin and Terman, 2004; Rubin et al., 2012). Note, optimal electrode placement for standard HF DBS might differ quite substantially from that of CR DBS. Computational results show that CR is effective no matter whether neurons are stimulated at somata or via inhibitory or excitatory synapses (Popovych and Tass, 2012). Accordingly, in our paper we concentrated on the central electrode placement, since from a clinical standpoint it might be feasible already with available depth electrodes.

Our investigations of different electrode positions showed that the placement of the stimulation electrode within the target structure is crucial for an effective desynchronization. Furthermore, the required stimulation current amplitude was dependent on the electrode location. In the context of a clinically more effective electrode placement it is still under discussion, whether the optimal stimulation site for HF DBS lies within the interior of the STN or in fiber tracts located close to the STN (Voges et al., 2002; Paek et al., 2011; Guo et al., 2012; Nakano et al., 2012; Reese et al., 2012). In order to tackle this issue utilizing a modeling approach, a future version of our model should include spatially extended multi-compartment neurons instead of pointlike model neurons. Hence, the mentioned fiber tracts should be represented by multi-compartment cable models.

Another feature of DBS relevant to possible further improvement is the shape of the stimulation pulses used. In various studies the efficacy of particular pulse shapes for neuronal stimulation was investigated (Kuncel and Grill, 2004; Kuncel et al., 2006; Butson and McIntyre, 2007; Foutz and McIntyre, 2010; Wongsarnpigoon and Grill, 2010; Hofmann et al., 2011). Apart from the stimulation pulse shapes, the other CR parameters (e.g., stimulation frequency, intra-burst frequency etc.) deserve further detailed investigations. Accordingly, our work provides a tool to probe related stimulation effects, which might be observed experimentally in future pre-clinical and clinical studies. Our model might contribute to a clinically useful computational tool for the optimization of parameters for CR DBS. In particular, the spatially extented topology of our model might be important for the development of new stimulation electrode geometries (Buhlmann et al., 2011; Martens et al., 2011).

### Conflict of interest statement

Peter Tass is employed by Jülich Research Center and works as Consulting Professor at Stanford University; formerly working with ANM GmbH (Cologne, Germany), shareholder of ANM GmbH. Christian Hauptmann is employed by Jülich Research Center; formerly working with ANM GmbH (Cologne, Germany). Several patents protect electrical CR neuromodulation. The inventor of this patent is Peter Tass, and the assignee is Jülich Research Center. Martin Ebert reports no potential conflicts of interest.
